# Psoriasis and staphylococcus aureus skin colonization in Moroccan patients

**DOI:** 10.11604/pamj.2016.23.33.7198

**Published:** 2016-02-08

**Authors:** Fatima Zahra Elfatoiki, Mohamed El Azhari, Assiya El Kettani, Zineb Serhier, Mohamed Bennani Othmani, Mohamed Timinouni, Hakima Benchikhi, Soumiya Chiheb, Hassan Fellah

**Affiliations:** 1 Department of Dermatology, Ibn Rochd UHC of Casablanca, Morocco; 2 Laboratory of Immunology, faculty of medicine, Casablanca, Morocco; 3 Laboratory of Microbiology, Pasteur Institute, Casablanca, Morocco; 4 Laboratory of Medical informatics, faculty of medicine, Casablanca, Morocco

**Keywords:** Antimicrobial peptides, innate immunity, nasal carriage, psoriasis, skin colonization, staphylococcus aureus

## Abstract

Psoriatic lesions are rarely complicated by recurrent infections. The aim of our study is to determine skin colonisation and nasal carriage of *Staphylococcus aureus* in patients with psoriasis and in healthy persons. Patients and methods: a comparative study that include 33 patients with psoriasis and 33 healthy persons. Samples were taken from lesional and non lesional psoriatic skin and from healthy skin of control group. For *S. aureus* nasal carriage, we used sterile cotton tipped swabs. Out of165 samples (66 skin samples and 33 nasal swabs), 26 *S. Aureus* strains were isolated in 26 persons, 57.69% in the control group and 42.3% in the psoriasisgroup. S. aureus skin colonization was found in one case (3%) inlesional psoriatic skin vs 9 cases (27.3%) in control skin OR=0.08 IC 95% (0.01-0.70) p=0.02 and in 12,1% in non lesional soriatic skin vs 27, 3% in control skin (p =0,13). This colonization was less important in lesional psoriatic skin (3%) than in non lesional psoriatic skin (12.1%) p= 0.20. Nasal screening identified (7/33) 21, 21% *S. aureus* carriers in psoriasis group and in control group. Our results are in consensus withliterature findings. They have confirmed the importance of antimicrobial peptides in Innateimmunity of human skin. These peptides are normally produced bykeratinocytes in response to inflammatory stimuli such as psoriasis. Their high expression in psoriasis skin reduces the risk of skin infection and skin colonization with *S. Aureus*.

## Introduction

Psoriasis is a chronic inflammatory skin disease which is thought to affect up to 5% of the world's population [1]. In the Maghreb, the prevalence of psoriasis was estimated at 12.08/1000 and in Morocco, it was estimated at 15.04/1000 [2]. This prevalence is probably underestimated due to the misunderstanding of the disease in our context [2]. For a long time, psoriasis has been thought to be an epidermal disease because of the increase in the keratinocytes proliferation rate and the shortening of the duration of their cell cycle [3]. However, current concepts of pathogenesis indicate the contribution of genetic, immunologic and environmental factors [4]. The presence of bacteria on and in the skin was also considered in the pathogenesis of psoriasis. There is also evidence that chronic plaques of psoriasis are related to streptococci in the throat [5]. Other microorganisms such as *S. Aureus* which usually resides on the skin, has also been implicated in the pathogenesis of psoriasis [6]. The plaques of psoriasis are rarely complicated by recurrent infections with bacterial, viral, and fungal pathogens. About 7 percent of patients with psoriasis have bacterial or viral skin infections, as compared with 30 percent of patients with atopic dermatitis [7]. Few studies were conducted to identify the colonising bacteria in psoriatic lesions [8]. The aim of our study is to compare skin colonisation and nasal carriage of *S. aureus* in patients with psoriasis vulgaris and in healthy persons.

## Methods

**Patients**: this is a comparative study performed in a university outpatient setting for 6 months. The study participants included 33 patients with psoriasis vulgaris and 33 healthy subjects. The inclusion criteria were patients with chronic plaque psoriasis aged over 16 years. None of the patients had received ultraviolet light or systemic therapies previously and none had received topical corticosteroids or antibacterial treatment for at least two weeks before enrollment. The study got approval from the Research and Ethical Committee of the Faculty of Medicine and Pharmacy of Casablanca, Hassan II University. The patients and healthy persons signed a written consent after being informed about the study.

**Collection of *S. aureus***: *S. aureus* skin colonization was obtained using Count-Tact Agar plates (Bio-Mérieux, Marcy l'Etoile, France). Specimens were collected in the psoriasis group from the psoriasis plaques and from non lesional skin of the back and in the control group from healthy skin of the back. For *S. aureus* nasal carriage, we used sterile cotton tipped swabs. The specimens were obtained by 5 rotations in each anterior nare of patients and healthy persons. All samples were quickly sent to the bacteriological laboratory.

**Isolation and identification of *S. aureus***: the studywasconductedat the Laboratory of Molecular Bacteriology of the Pasteur institute, Casablanca, Morocco. The Count-Tact Agar plates were incubatedaerobicallyat 36°C for 24 to 48 h. The nasal swabs were inoculated on Chapman agar (local production according to its composition), and incubated at the same conditions. After incubation, The colonies were identified on the basis of their morphologies, mannitol fermentation, catalase test and Gram staining, coagulase activity on rabbit plasma (Bio-Mérieux, Marcy l′Etoile, France), production of clumping factor (Pastorex Plus-Staph, Bio-Rad, Marnes-la-Coquette, France) and biochemical features using theApi Staph Bio-Mérieux France microorganism identification test kit. The homogeneous strains were stored at 4°C in nutrient agar until use, and at -20°C in glycerol stocks using commercial Cryobilles (AES Laboratoire, France).

**Antimicrobial resistance**: Antibiotic sensitivity of *S. aureus* strains was obtained using the agar disc diffusion method on M-H medium (Bio-Rad, France) according to the recommendations given by the CA-SFM (2012). The following antimicrobial agents (Bio-Rad, France) were tested: cefoxitin (30 μg), penicillin G (6 μg), tetracycline (30 μg), erythromycin (15 μg), lincomycin (15 μg), pristinamycin (15 μg), kanamycin (30 μg), tobramycin (30 μg), gentamicin (10 μg), chloramphenicol (30 μg), rifampicin (5 μg), fosfomycin (50 μg), pefloxacin (5 μg), trimethoprim/sulfamethoxazole (1.25/23.75 μg), fusidic acid (10 μg) and vancomycin (30 μg). S. aureus isolates were considered as multidrug resistant (MDR) when they were resistant to 3 or more of the antibiotics listed above.

**Methicillin sensitivity**: Methicillin susceptibility was studied by using agar screen plates on Muller-Hinton (M-H) agar (Bio-Rad, France) with 30 μg/ml of cefoxitin disc as recommended by the French Microbiological Society Antibiogram Committee (CA-SFM 2012). Inhibition diameter around cefoxitin disks less than 27 mm after incubation at 36°C for 18-24 h. reflects MRSA suspicion. MRSA U2A1593 and methicillin-susceptible *S. aureus* U2A1594 provided by Pasteur Institute (Paris, France) were used as controls.

**Statistical Analysis**: Data was analyzed using SPSS statistical software, version 16.0 (IBM, Chicago, USA). Chi-Square test was used to assess the differences between proportions of *S. aureus* strains isolated in psoriasis lesional skin, psoriasis non-lesional skin and control skin. Odds Ratio was calculated to assess the association between *S. aureus* skin colonization and psoriasis. A (p-value <0.05) was considered as statistically significant.

## Results

Out of 33 psoriasis patients 10 were women (30. 3%) and their mean age at the time of enrollment was 43. 21(SD=15.80). Of the 33 control individuals, 14 were women (42, 4%) and their mean age at the time of enrollment was 38. 94 (SD=14. 35). Diabetes was found in 3 patients in the psoriasis group versus 2 persons in the control group. In the 165 samples (66 skin samples and 33 nasal swabs) obtained from 66 subjects, a total of 26 *S. aureus* strains was isolated in 26 persons, 15/26 (57. 69%) in the control group and 11/26 (42. 3%) in the psoriasis group (Table 1).

**Table 1:** Distribution of S. aureus isolated in psoriasis and control groups

	Psoriasis group=11(42,3%)			Control group=15(57,69%)	
	Nas. carriage	NLPS	LPS	Nas. carriage	control skin
S. aureus isolated	7	4	1	7	9

NLPS : non lesionalpsoriatic skin/ LPS : lesionalpsoriatic skin. In psoriasis group one S. aureus strainwasfound in both NLPS and nasal carriage and in control group one S. aureus strainwasfound in both control skinand nasal carriage

**Cutaneous colonization**: S. aureus skin colonization was found in one case (3%) in lesional psoriatic skin versus 9 cases (27.3%) in control skin OR=0.08 IC_95%_ (0.01-0.70) (p=0.02) and in 4 cases (12.1%) in non lesional psoriatic skin versus 9 cases (27. 3%) in control skin OR=0.37 IC_95%_ (0.10-1.34) (p =0.13). This colonization was less prevalent in lesional psoriatic skin (3%) than in non lesional psoriatic skin (12.1%) OR=0.23 IC_95%_ (0.02-2.15) p= 0.20. Bacterial load of *S. aureus* was more important in control skin. Intense bacterial load of *S. aureus* (> 300 colonies) was not found in lesional psoriatic skin however it was found in 3% in non lesional psoriatic skin and in 9.1% in control skin, but these associations were not significant too.

**Nasal carriage**: Nasal screening identified 21, 21% *S. aureus* carriers in psoriasis group and 21, 21% *S. aureus* carriers in control group.

**Antibiotic susceptibility**: A total of 10 various resistance phenotypes were found for the 26 *S. aureus* strains isolated (Table 2). The control skin isolates showed the highest number of resistance (Table 3). Wild-type phenotype (*S. aureus* susceptible to all antimicrobial agents tested) was found in one nasal carriage in the psoriasis group and Methicillin resistant *S. aureus* (MRSA) was found in three cases (12%) in control skin. Multi-resistance to three antibiotic classes or more was not observed in lesional psoriatic skin isolates while 1 (3%) and 2 (6.1%), Multi-resistant *S. aureus* were found respectively in non lesional psoriatic skin and in control skin. The difference was not statistically significant.

**Table 2:** Resistance phenotype profile of S. aureus strains isolated in psoriasis and control groups

Resistance phenotype profile	Frequency in psoriasis group n(%)	Frequency in control group n(%)
PG	7 (21,21%)	7(21,21%)
FA	1(3,03%)	0
PG,TE	0	2(6,06%)
PG, FA	1(3,03%)	0
PG, Pef	0	1(3,03%)
PG, E	0	2(6,06%)
PG, Fox	0	1(3,03%)
PG,FA, C, Sxt, E, RF	1(3,03%)	0
PG,Fox, Pef, FA, C, L, RF	0	1(3,03%)
PG, Fox, FA, C,L, RF	0	1(3,03%)

**Table 3:** S. aureus antibiotic suscepibility in psoriatic and control groups

Antimicrobial	psoriasis group n(%)	control group n(%)
PG	9 (27,27%)	14(42,42%)
FA	3(9,09%)	2 (6,06%)
TE	0	2(6,06%)
E	0	2(6,06%)
Pef	0	2(6,06%)
Fox	0	3(9,09%)
Sxt	1(3,03%)	0
RF	1(3,03%)	2(6,06%)
C	1(3,03%)	2(6,06%)
L	0	2(6,06%)

## Discussion

In our study, we have revealed that the percentage of *S. aureus* colonization was significantly higher in the control skin than in the lesional psoriatic skin (27.3% vs 3%) OR=0.08 IC_95%_ (0.01-0.70) (p=0.02). Thus, the psoriasis is a protective factor against skin *S. aureus* colonization. This difference was already reported by Fahlen et al [8] in 2012 who studied the distribution of bacterial microbiota in normal and psoriatic skin. In that study, this bacterial microbiota was more important in normal skin compared with psoriatic skin. Moreover, Staphylococcus in both limbs and trunk was more abundant in control group skin compared with psoriasis group skin. They suggested that this difference was disease-related and that the psoriasis could protect against bacterial colonization. In another study [9] comparing *S. aureus* infection and antimicrobial peptides in patients with atopic dermatitis, psoriasis and in normal subjects; the authors highlighted the role of antimicrobial peptides in innate immunity of human skin and in the ability of the skin to resist bacterial infection. They explained that Cathelicidins (LL-37) and defensins (HBD-2) belonging to this antimicrobial peptides family contribute to hostdefense against *S. aureus*.

The results of this study showed significantly lower expression of HBD-2 mRNA and LL-37 mRNA in atopic lesions than in psoriatic lesions (P=0.009 and P=0.02, respectively). The combination of LL-37 and HBD-2 showed synergistic antimicrobial activity by effectively killing *S. aureus*. They concluded that a deficiency in the expression of antimicrobial peptides may account for the susceptibility of patients with atopic dermatitis to skin infection with *S. aureus*. The antimicrobial peptides HBD-2 and LL-37 are normally produced by keratinocytes in response to inflammatory stimuli such as psoriasis or injury [10]. Their high expression in psoriasis skin reduces the risk of skin infection and skin colonization with *S. aureus* [9]. During the study, we have also found that *S. aureus* colonization was less important in non lesional psoriatic skin (12.1%) than in control skin (27.3%) however the difference was not significant (p= 0.13). It has recently been reported that the skin flora varies depending on the site from which the samples are taken [11, 12]. The authors divided the body surface into three groups: sebaceous, moist and dry and found that bacterial load varied within these skin sites [11, 13]. However, in our study, all skin biopsies of non lesional psoriatic skin and control skin were taken from the back. Thus, the difference between *S. aureus* skin colonization in non lesional psoriatic skin and control skin is not related to the site of skin biopsy, but rather to antimicrobial peptides production. These results are in consensus with findings by Peck and al [9] who confirmed the absence of HBD-2 and LL-37 in normal skin compared with psoriatic skin. These antimicrobial peptides are up-regulated under inflammatory conditions such as psoriasis [10, 13].

Thus in our study, HBD-2 and LL-37 could be present in non lesional psoriatic skin since psoriasis is an inflammatory disease with a production of systemic inflammatory cytokines. These cytokines could lead to increased synthesis of antimicrobial peptides by keratinocytes even in non lesional psoriatic skin but in low rates. This hypothesis could explain the poor colonization with *S. aureus* in non lesional psoriatic skin (12.1%) compared with control skin (27.3%) and in lesional psoriatic skin (3%) compared with non lesional psoriatic skin(12.1%). Moreover, intense bacterial load was more important in control skin followed by non lesional psoriatic skin then by lesional psoriatic skin. This difference may be related to a local production of antimicrobial peptides which is more important in psoriatic skin [9]. *S. aureus* nasal carriage rate in the present study was found to be 21, 21% in both psoriasis group and control group. These findings are consistent with figures that usually quoted for *S. aureus* nasal carriage of 20-40% [14]. When assessing the susceptibility to antibiotics of the 26 *S. aureus* strains, the control group showed the highest number of resistance. MRSA was found in 12% of isolates, all originating in control group skin. Multi-resistance to three antibiotic classes or more was not observed in lesional psoriatic skin isolates compared with 3% and 6.1% multi-resistant *S. aureus* respectively in non lesional psoriatic skin and in control skin. The difference was not statistically significant. Further studies are required to determine genetic characteristics of *S. aureus* strains isolated.

## Conclusion

Our study suggests that skin *S. aureus* colonization from control and psoriasis groups shows some differences. However, the number of samples was very limited and further work is needed to investigate these possible differences and to compare the expression of antimicrobial expression in different sites.

### What is known about this topic

- Psoriasis lesions are rarely complicated by recurrent infections.

### What this study adds

- *S. aureus* colonization was more important in normal skin of control group compared with psoriatic skin.
- *S. aureus* colonization was more important in non lesional psoriatic skin compared with lesional psoriatic skin of the same patients.
